# The gut microbiota-inflammation-brain axis in end-stage renal disease: perspectives from default mode network

**DOI:** 10.7150/thno.35387

**Published:** 2019-10-18

**Authors:** Yun Fei Wang, Li Juan Zheng, Ya Liu, Yu Bing Ye, Song Luo, Guang Ming Lu, Dehua Gong, Long Jiang Zhang

**Affiliations:** 1 Department of Medical Imaging, Medical Imaging Center, Jinling Hospital, Medical School of Nanjing University, 305 Zhongshan East Road, Xuanwu District, Nanjing, Jiangsu Province, 210002, China.; 2 National Clinical Research Center of Kidney Disease, Jinling Hospital, Medical School of Nanjing University, 305 Zhongshan East Road, Xuanwu District, Nanjing, Jiangsu Province, 210002, China.

**Keywords:** end-stage renal disease, gut microbiota, default mode network, inflammatory cytokine, resting state fMRI

## Abstract

The gut-brain axis in end-stage renal disease (ESRD) is attracting more and more attention. However, the mechanism of gut-brain axis based cognitive disorders in ESRD patients remains unclear. The purpose of this study was to investigate the linkages between the gut microbiota, inflammatory cytokines, brain default mode network (DMN) and cognitive function in ESRD patients.

**Methods:** This prospective study enrolled 28 ESRD patients (13 males and 15 females, mean age of 44 ± 14 years) and 19 healthy controls (HCs) (12 males and 7 females, mean age of 44 ± 10 years). All subjects underwent stool microbiota analysis, blood inflammatory cytokines examination, brain MRI scans and cognitive assessments. Resting state functional MRI (rs-fMRI) data were used to construct DMN and graph theory was applied to characterize network topological properties. Two samples t-test was applied for the comparisons between ESRD and HCs. Correlation analysis and mediation analysis were conducted among factors with significant group differences.

**Results:** ESRD patients displayed gut microbiota alterations, increased systemic inflammation and worse cognitive performance compared to HCs (all p < 0.05). Graph analysis revealed disrupted DMN topological organization, aberrant nodal centralities and functional connectivities (FCs) in ESRD patients relative to HCs (all p < 0.05, FDR corrected). Significant correlations were found between gut microbiota, inflammatory cytokines, DMN network measures and cognitive assessments. Mediation analysis found that gut microbiota alteration impaired DMN connectivity by increasing systemic inflammation.

**Conclusion:** The present study first revealed gut microbiota alterations, systemic inflammation, DMN dissociation and cognitive dysfunction in ESRD patients simultaneously and further illuminated their inner relationship.

## Introduction

Chronic kidney disease (CKD) is a widespread public health problem, notably accompanied by various cognitive disorders 1,2. Particularly, patients with end-stage renal disease (ESRD) suffered from high rates of neurocognitive dysfunction 3. This neural complication seriously affected patients' daily life as well as clinical treatment, which may lead to poor clinical outcomes 4-6. However, the neuro-mechanism of the cognitive disorders in ESRD patients remains unclear. Hence, to understand the potential neural pathways and find new therapeutic target are of vital importance.

Among the identifiable resting state brain networks, default mode network (DMN) is the most consistent one 7,8. Converging findings have suggested that DMN is involved in the core processes of brain function (such as internal mentation, attention, and monitoring of the surrounding environment) 9. Disruption of the DMN has been detected in some neuropsychological disorders 10,11 as well as ESRD 12,13. However, these previous studies only focused on the spontaneous regional activity or the functional connectivity 14,15 in ESRD patients, which did not assess the crucial topological configuration of the DMN. Growing evidence has suggested that brain disorders are associated with the altered topological architecture of brain network. Graph theory is an advanced method that enables us to investigate the topological pattern of complex brain network and further reveal the potential neuro-mechanism of the cognitive disorders 16.

Recently, the concept of “gut-brain axis” has been proposed and hint gut microbiota could shape the brain 17-20. Animal studies have also proved that gut microbiota changes could mediate anxiety- and depression-like behavioral alterations of mice 21. Some studies have emphasized that human gut microbiota plays an important role in the pathogenesis and development of CKD 22-24. Some studies also found the relationship between gut microbiota and inflammatory status in the context of CKD 25. However, how the gut affects the brain in patients with CKD is unclear. Considering the potential effect of inflammatory status on the brain in recent studies 26-28, we hypothesized that the gut microbiota may cause central nervous system disorders via systemic inflammation in ESRD patients.

In the current study, we implemented graph theory approach to systematically investigate DMN alterations in ESRD patients. Moreover, we sought to further reveal the underlying relationships between gut microbiota, inflammatory status, DMN patterns and cognitive function in ESRD patients.

## Material and Methods

### Participants

This prospective study was approved by the local Medical Research Ethics Committee. Twenty-eight ESRD patients were enrolled from the Department of Nephrology, Jinling Hospital and 19 age-, gender- and education level-matched healthy controls (HCs) were also recruited. All subjects were right-handed. ESRD patients were included with the following criteria: estimated glomerular filtration rate (eGFR) < 15 ml/min/1.73m^2^ or undergoing dialysis treatment. The inclusion criteria for HCs: 1) could finish MR examination and cognitive assessments; 2) did not take any antibiotics, probiotics or catharsis drugs. The exclusion criteria were: 1) history of brain tumor, brain trauma, neuropsychiatric disorders; 2) obvious diarrhea, constipation or intestinal inflammation; 3) alcohol addiction or drug abuse history; 4) contraindication of MR examination; 5) immune-mediated inflammatory disease (IMID) such as Crohn's disease or ulcerative colitis.** Table 1** shows the demographical, inflammatory cytokine and neuropsychological data of all subjects.

### Blood Samples Collection and Analysis

Peripheral blood was collected from each participant to evaluate the value of plasma inflammatory cytokines on the day of fecal specimen collection. The inflammatory cytokines include interleukin-6 (IL-6) (pg/mL), interleukin-1alpha (IL-1alpha) (pg/mL), interferon-gamma (IFN-gamma) (pg/mL) and tumor necrosis factor-alpha (TNF-alpha) (pg/mL). The blood biochemical indicators (which included urea nitrogen (mg/dl), creatinine (mg/dl), uric acid (μmol/L) were also examined for each ESRD patient.

### Fecal Samples Collection and Intestinal Flora Analysis

Fecal samples were collected within 2 days before or after MR examination. The fresh middle section of the stool sample was obtained for avoiding contamination, and were stored at -80℃ immediately after collection. First, DNA samples were quantified and the V3-V4 hypervariable region of 16S rRNA was selected for PCR amplification. Next, the raw reads were filtered and then compared with the Gold database. Each OTU representative sequence was selected for species annotation and classification. The Alpha diversity Shannon index was also calculated. The detailed intestinal flora analysis procedures are available in the **Supplementary Materials**.

### Neuropsychological Assessments

All participants underwent a battery of standardized neuropsychological assessments before MRI scanning. Global cognitive function was measured by the Mini-Mental State Examination (MMSE) and Montreal Cognitive Assessment Scale (MoCA) 29. Information processing speed was derived from the Digit Symbol Test (DST) 30). Psychomotor speed was derived from Line Tracing Test (LTT), Serial Dotting Test (SDT) and Number Connection Test type-A (NCT-A). Depression and anxiety state were measured using the Self-Rating Depression Scale (SDS) 31 and Self-Rating Anxiety Scale (SAS) 32.

### MRI Data Acquisition and Preprocessing

All MRI data were acquired in a 3.0-T MR scanner (Discovery MR 750; GE Medical Systems, Milwaukee, WI, USA) using a 32-channel phased-array head coil. First, T2 fluid-attenuated inversion-recovery (T2-FLAIR) sequence was conducted to exclude the clinically silent lesions. Then, the rs-fMRI data were collected using single-shot, gradient-recalled echo-planar imaging sequence. Next, high-resolution three-dimensional T1-weighted images (3D-T1WI) data were obtained using magnetization-prepared rapid gradient-echo (MPRAGE) sequence. Comfortable fixed foam pads were used to reduce head movements and ear plugs were used to minimize scanner noise. During the rs-fMRI scan, all participants were instructed to relax, keep their eyes closed and avoid thinking anything in particular. Image preprocessing was conducted using the Data Processing & Analysis Assistant for Resting-State Brain Imaging (DPABI) 33 and Statistical Parametric Mapping 8 (SPM8, http://www.fil.ion.ucl.ac.uk/spm). The detailed scanning parameters and preprocessing steps are listed in the **Supplementary Materials**.

### DMN Construction

**Figure 1:**
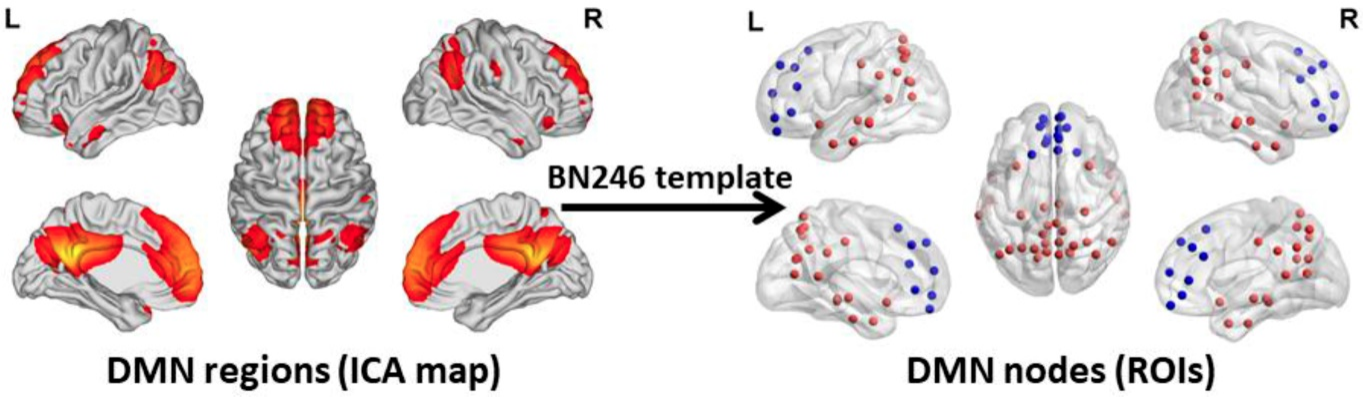
** DMN regions and DMN Nodes.** The graphs depict the 50 DMN nodes derived from the functional atlas (BN246) that were used for the primary analysis. The picture in the left side shows the DMN map and the picture in the right side shows the ROI selected according to the DMN map. The blue ROIs represent the regions belong to the anterior DMN and the red ROIs represent the regions belong to the posterior DMN. The picture is made using the BrainNet Viewer software (http://www.nitrc.org/projects/bnv). DMN: default mode network; ICA: independent component analysis; ROI: region of interest.

To acquire an unbiased DMN map for each subject, spatial independent component analysis (ICA) was performed using the Group ICA 2.0 of fMRI Toolbox (GIFT, http://www.icatb.sourceforge.net). The anterior DMN and posterior DMN were identified in the resting state condition at the group level. The anterior and posterior DMN maps were merged together to create a complete DMN map. Next, the DMN nodes were selected according to the group-level DMN map. In this study, we chose a more fine-grained functional brain atlas (BN246 Atlas). This atlas was constructed by using more detailed anatomical and functional connection patterns, which could more accurately describe the locations of the activation or connectivity in the brain 34. The nodes of the DMN are shown in **Figure 1**.**Table S1** illustrates the detailed information of the BN246 template and DMN nodes selected in this study.

### Graph-theory Analysis

For graph-theory analysis, the mean time series for each voxel within the node of the DMN were extracted. Next, Pearson correlation coefficients were computed between each pair of DMN nodes to generate a 50×50 correlation matrix. Then, we used the graph theoretical network analysis (GRETNA) toolbox 35 to evaluate the topological organization of the DMN. We calculated the network topological properties at (1) global level, which included clustering coefficient (Cp), characteristic path length (Lp), normalized clustering coefficient (Gamma), normalized characteristic path length (Lambda), small-worldness (Sigma), global efficiency (Eg) and local efficiency (Eloc); (2) nodal level, which included nodal degree, nodal efficiency and nodal betweeness; (3) edge level, which included all the functional connectivities between each pair of DMN nodes. **Table S2** gives detailed descriptions of the above-mentioned graph measures. Additionally, we calculated another global level network measure: anterior DMN to posterior DMN connectivity (aDMN-pDMN). Since we obtained both the anterior and posterior map of the DMN, we calculated the FC between the anterior DMN and posterior DMN and defined it as aDMN-pDMN. We considered it as an important DMN parameter and some previous studies have also found the important role of aDMN-pDMN in normal aging process 36 and disease condition 37.

We operated network parameters over a range of threshold values to guarantee high correlation coefficients of the remaining connections. We chose a sparsity threshold (range from 0.08 to 0.4 with an interval of 0.01) to convert each of the resulting correlation matrices into a series of weighted networks 38,39. We further calculated the area under the curve (AUC) over the sparsity for the between-group comparisons. The AUC provides a summarized scale for the topologic characterization of brain networks, which is sensitive to topologic alterations in brain disorders 40.

### Total Intracranial Volume Calculation

Considering the potential effect of brain volume on brain network analysis results, we analyzed the total intracranial volume (TIV) differences among the two groups (**Table 1**). The analysis steps are consistent with our previous article 41.

### Statistical Analysis

Statistical analysis of demographic, neuropsychological and clinical data was performed using SPSS software (IBM SPSS Inc., Chicago, IL, USA). Student's t test was used to compare the continuous variables and χ2 test was used to compare categoric variables. Before the analyses were performed, the Kolmogorov-Smirnov test was used to test for normality for quantitative data. Normally distributed data were presented as mean ± standard deviation and then assessed by independent two sample t-test. Mann-Whitney U tests were used to analyze the data with non-normal distribution, which were reported as median and inter-quartile range. For the analysis of microbiota, Mann-Whitney U test was used to assess the differences between groups because of the non-normally distributed data. The species difference analysis procedures of gut microbiota were supplied in the **Supplementary Materials**.

Between-group differences of network measures were determined by independent two sample t-test in the GRETNA toolbox. Age, sex, educational level, body mass index (BMI), TIV and emotional scale scores were set as covariates to exclude the potential effects. Furthermore, correlation analysis was performed between each two factors, which were derived from the significant differences of the gut microbiota, inflammatory cytokines, DMN parameters and cognitive scale scores between the two groups. Pearson correlation was applied for the normally distributed data and the Spearman correlation was applied for the non-normally distributed data. Correlation network was constructed by the factors with the significant correlation in all the subjects. P < 0.05 was considered statistically significant and false discovery rate (FDR) correction 42 was performed for multiple comparisons in all the above-mentioned analysis.

However, correlation analysis only provides pairwise associations among the gut, inflammation and brain in this study, an entire “gut-inflammation-brain” pathway remains unclear. A mediating pathway is very useful for developing directional/causal hypothesis under this circumstance 43. We employed mediation analysis to determine whether the systematic inflammation acted as a mediating role in the effects of gut microbiota on DMN topological patterns in ESRD patients. In the mediating model, the gut microbiota, systematic inflammation, and DMN topological patterns were established as the predictor, mediator, and outcome, respectively. Considering the microbiome and inflammation may be bi-directional, we also tested another mediation pathway (inflammation as predictor and microbiome as mediator) in this study. The entire mediation analysis was performed using the PROCESS macro implemented in SPSS 44. The mediation was calculated based on two linear mixed effect (LME) models as demonstrated below:


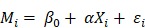



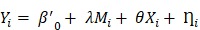


Here 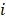 denotes subject (gut microbiota alteration in the present study). 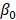 and 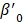 are the intercepts for M and Y, respectively. The effect of X on M is designated as 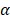, the effect of M on Y is designated as 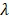, and the direct effect of X on Y is designated as 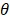. 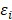 and 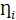 are residuals for M and Y, respectively. Next, 5000 bootstrap samples were generated to estimate the bootstrap confidence intervals (CIs) for the indirect effect. An empirical 95% confidence interval (CI) that did not include zero indicated that the indirect effect was significant at the p < 0.01 level 45. For detailed analysis steps, please see **Supplementary Materials**.

### Reliabilities of Network Measures

Recognizing the potential variability due to the choice of atlas, we repeated the DMN topological analyses using another functional brain atlas (Power 264 atlas) 46 to test the reliabilities of DMN analysis.

## Results

### Demographical, Neuropsychological and Inflammatory Cytokine Results

There were no significant inter-group differences in terms of age, sex or education level (all p > 0.05). In neuropsychological assessment, ESRD patients performed worse in MoCA, NCT, SDT, DST, SAS and SDS compared with HCs (all p < 0.05). Additionally, the ESRD patients showed higher IL-6, IFN-gamma and TNF-alpha (all p < 0.05) than HCs. Demographic, neuropsychological assessment, and inflammatory cytokine results are shown in **Table 1.**

### DMN Changes between ESRD Patients and HCs Differences in Global Network Measures of the DMN

**Figure 2:**
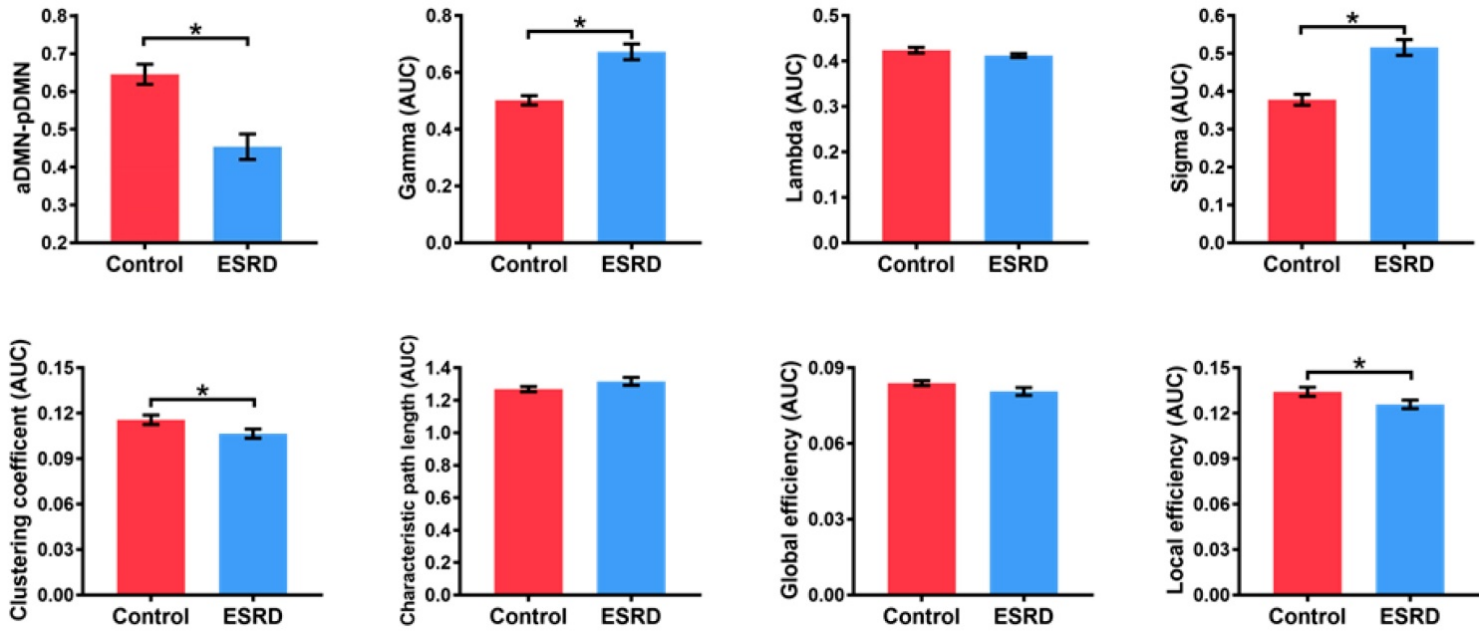
** Differences in Global Network Measures of the DMN.** The graphs show global network measures of the DMN. The red histogram represents healthy controls and the blue histogram represents ESRD patients. Black asterisks (*) indicate the significant difference between groups (p < 0.05). AUC: area under the curve; DMN: default mode network; ESRD: end-stage renal disease.

Compared with HCs, ESRD patients showed weaker aDMN-pDMN connectivity (p = 0.001). Over the entire range of thresholds (from 0.08 to 0.40), both ESRD patients and HCs exhibited small-world properties (Gamma obviously larger than 1 and Lambda close to 1) of the DMN. However, compared to HCs, ESRD patients showed significantly increased Gamma (p = 0.003) and Sigma (p = 0.001) as well as decreased Cp (p = 0.011) and Eloc (p = 0.024). In contrast, there were no significant intergroup differences in Lambda (p = 0.131), Lp (p = 0.110) or Eg (p = 0.078) (**Figure 2**). Detailed global network measures are illustrated in **Table 2**.

### Differences in Nodal Network Measures of the DMN

**Figure 3:**
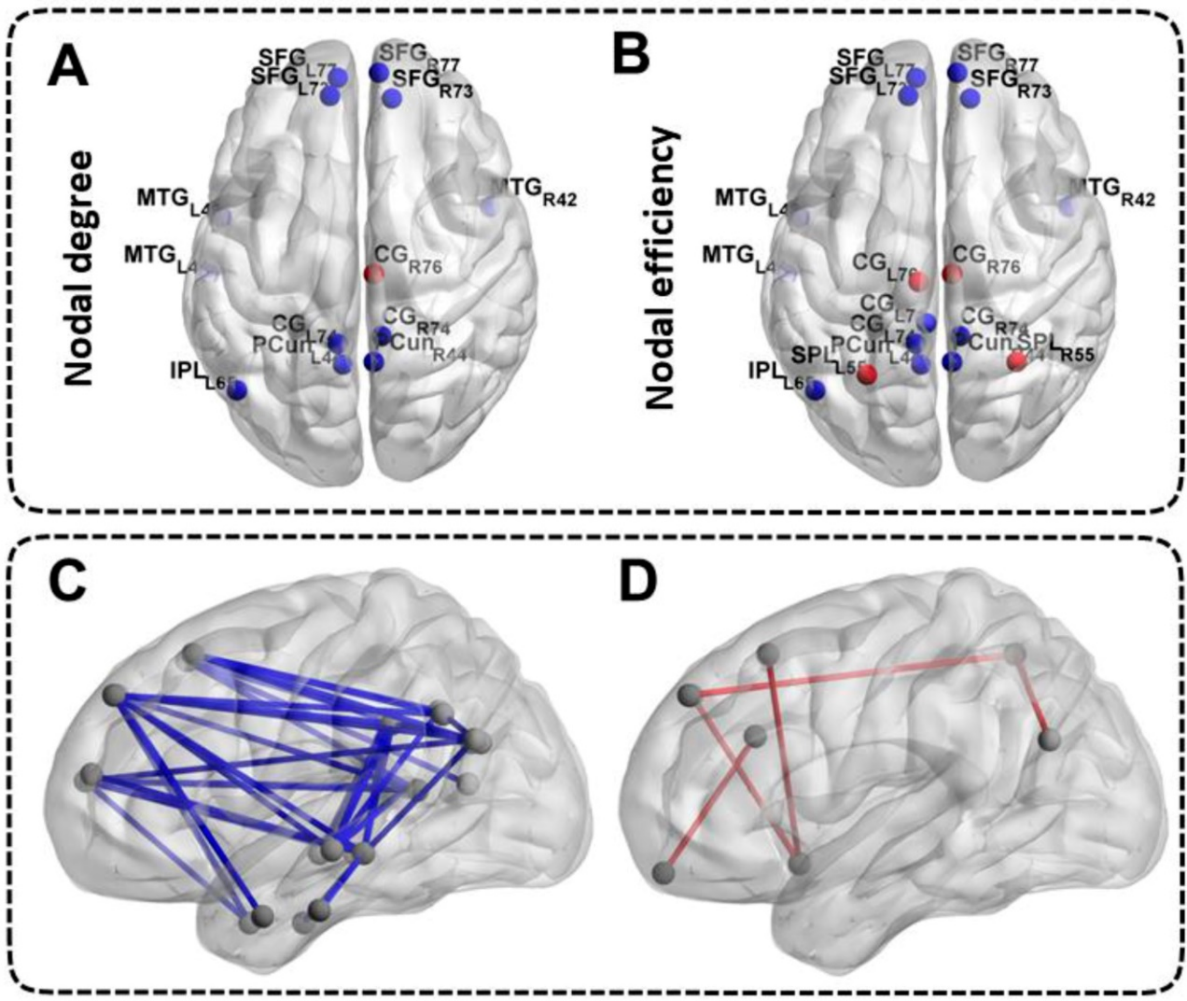
** Differences in Nodal Network Measures and Functional Connectivity Measures of the DMN.** The top half of the picture represents the differences in nodal network measures and the bottom half of the picture represents the differences in functional connectivity measures. The blue nodes and blue lines indicate decreased nodal centralities and functional connectivities. The red nodes and red lines indicate increased nodal centralities and functional connectivities. The abbreviations of the nodal label were explained in the **Table S1**.

Patients with ESRD manifested abnormal nodal centralities, which showed significant between-group differences in at least one nodal metric, including nodal efficiency and nodal degree. Compared with the HCs, ESRD patients showed decreased nodal degree and nodal efficiency in the bilateral SFG, MTG, CG, PCun and the left IPL (all p < 0.05, FDR-corrected). Moreover, ESRD patients also exhibited increased nodal degree in the right CG, and increased nodal efficiency in the bilateral CG and SPL (all p < 0.05, FDR-corrected) (**Figure 3A, Figure 3B**). No significant differences in nodal betweenness were observed among the two groups. The description of the node label of the DMN could be found in**Table S1**.

### Differences in Functional Connectivity Measures of the DMN

A large amount of decreased functional connectives (mainly between anterior DMN and posterior DMN) were found in ESRD patients compared with HCs (all p < 0.05, FDR-corrected) (**Figure 3C**). However, a small number of increased functional connectives were also found in ESRD patients (mainly between the SFG, INS, ITG, CG, IPL, SPL, MTG and OrG) compared to HCs (all p < 0.05, FDR-corrected) (**Figure 3D**). The description of the node label of the DMN could be found in**Table S1**.

### Analysis of Intestinal Flora in Patients with ESRD

The histogram of the community composition of the genus level showed that both the ESRD and the HC groups were mainly Bacteroides. Specifically, the Prevotella, the Faecalibacterium and the Fusobacterium displayed relatively high abundance in the ESRD group, while the abundance of the Megamonas and the Clostridium were relatively high in the HC group (**Figure S1A**). By using the LEfSe differential analysis based on the all levels of species abundance, it was found that the ESRD group was mainly enriched in the Phascolarctobacterium, while HC group was mainly enriched in the Roseburia, Megasphaera, Peptostreptococcaceae, Dialister, Lachnospira, Bifidobacteriales, Bifidobacteriaceae, Bifidobacterium, Bacillaceae, Bacillus and Actinobacteria (**Figure S1B**). Furthermore, the Wilcox differential analysis based on genus-level species abundance revealed that the ESRD group was mainly enriched in Phascolarctobacterium, Holdemania and Eggerthella, while mainly decreased in Roseburia, Lachnospira, Dialister and Bifidobacterium (**Figure S1C**). Additionally, the differences of intestinal flora alpha diversity between the ESRD group and HC group was not significant (p = 0.497) according to the Shannon index (**Figure S1D**).

### Correlation Networks of Gut Microbiota, Inflammatory Cytokines, Brain Network Measures and Cognitive Assessments

**Figure 4:**
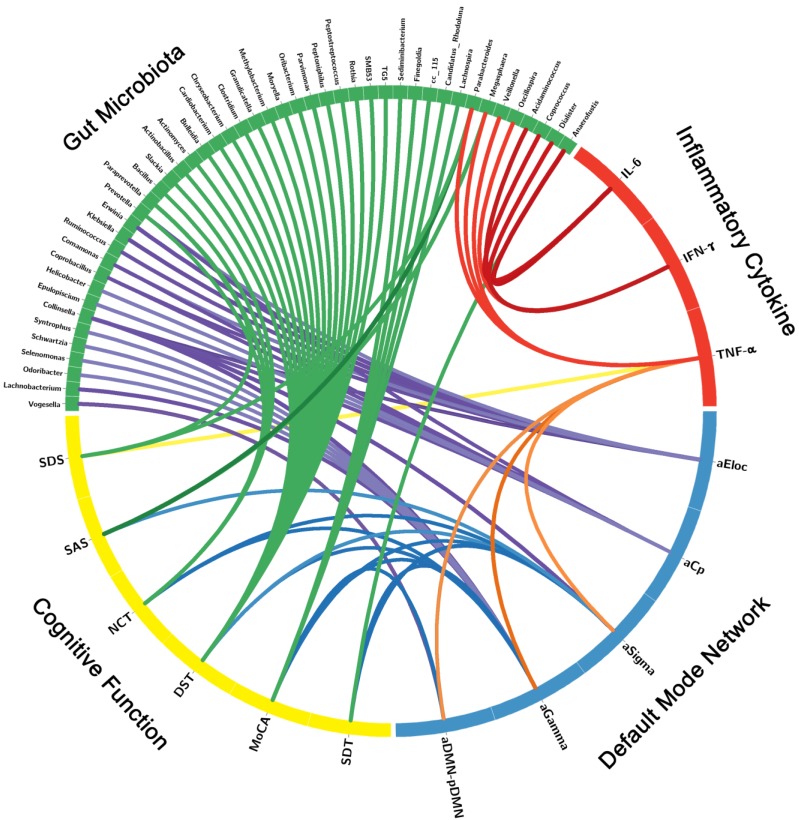
** Correlation Networks of Gut Microbiota, Inflammatory Cytokines, Cognitive Assessments and Brain Network Parameters.** Graph shows correlations between gut microbiota, inflammatory cytokines, cognitive assessments and DMN parameters with group differences. Green block represents gut microbiota; yellow block represents cognitive function; blue block represents default mode network; red block represents inflammatory cytokine. Link transparency encodes the r value: the lower color opacity corresponds to the smaller absolute r value of the correlation; and the higher opacity corresponds to the larger absolute r value of the correlation.

The correlation network of gut microbiota, inflammatory cytokines, DMN topological measures and cognitive assessments were constructed by the factors with significant correlation. Significant correlation was defined as r > 0.3 and p < 0.05 after FDR correction (**Figure 4**, **Table S3**). The correlation network showed that these factors correlated with each other, which indicated the inner association in this circuit.

### The Mediation of Systematic Inflammation in the Effect of Gut Microbiota on DMN Topological Architecture in ESRD Patients

**Figure 5:**
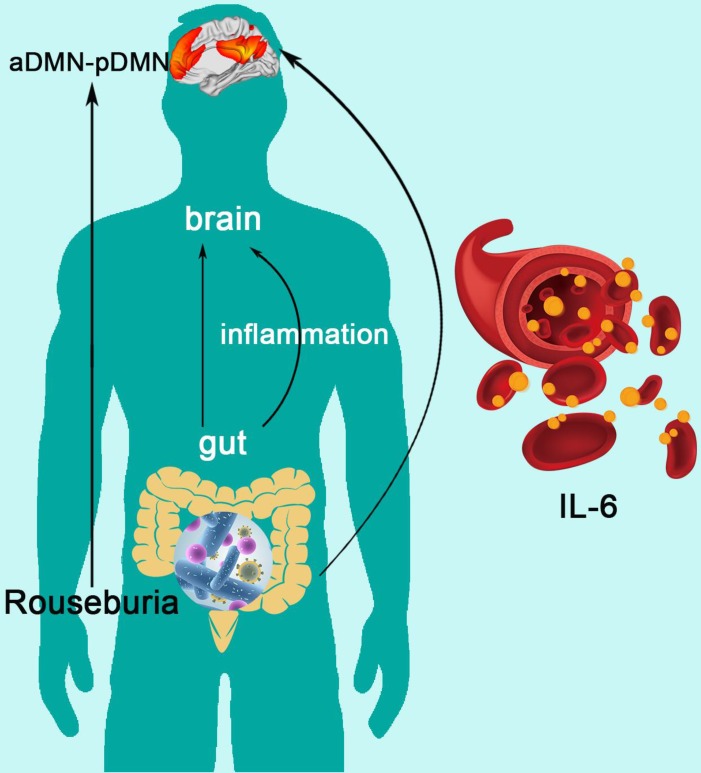
The Mediation of Systemic Inflammation in the Effect of Gut Microbiota on DMN Topological Architecture in Patients with ESRD. Mediation analysis demonstrates that IL-6 mediates the relationship of Roseburia and aDMN-pDMN connectivity corrected with age and sex (p < 0.001, β = -4.156, 95%CI: [-22.249, 19.258]). DMN: default mode network; IL-6: interleukin-6.

Mediation analysis showed that the relative abundance of Roseburia was associated with aDMN-pDMN connectivity (total effect: β = 33.245, p=0.045), and there was a significant mediation effect by IL-6 (indirect effect: p < 0.001, β = -4.156, 95%CI: [-22.249, 19.258]) for the association between Roseburia on aDMN-pDMN. After taking into consideration of the significant path via IL-6, the direct effect of Roseburia on aDMN-pDMN was also significant (β = 37.401, p=0.045) (**Figure 5**). For the mediation pathway with inflammation as predictor and microbiome as mediator, we did not find any statistically significant result (all P>0.05).

### Reliabilities of Network Measures

The calculations of the network measures using the different node definitions (Power 264 atlas) revealed a largely similar pattern of results, which further confirmed the reliability of our results. Details can be found in **Figures S2-4, Table S4**.

## Discussion

This prospective study of “gut-inflammation-brain axis” investigated topological changes of DMN in ESRD patients and further explored the potential relationship with gut microbiota and inflammation cytokines. The results revealed that IL-6 mediated the effect of Roseburia on aDMN-pDMN connectivity, indicating that the alterations of gut microbiota impaired DMN topological architecture by increasing systemic inflammation. To the best of our knowledge, this is the first study on the association of functional MRI based brain default mode network, cognitive function, gut microbiota and inflammatory state. These findings may provide novel insights into the new therapeutic target using probiotics in ESRD-induced cognitive disorders.

In the network analysis, both patients and controls showed economic small-world properties in the DMN. Although the DMN has a small-world organization, our results identified higher Gamma and Sigma values in ESRD patients, hinting that the DMN in individuals with ESRD shows topological alterations. In addition, lower DMN Cp and Eloc were detected in ESRD patients relative to HCs. Cp is a network measure that quantifies the strength of network segregation 47 and Eloc predominantly reflects the capacity of regional information transmitting 48. The combination of low Cp and Eloc reflects a low local specialization of information processing in the network. Namely, our findings revealed the disconnections between adjacent brain regions in the DMN. Moreover, normalized Cp was correlated with the cognitive assessment scores (MoCA, NCT, DST and SDT), which further illustrated that disrupted topology architecture of the DMN may underlie the cognitive disorders in patients with ESRD. We also observed decreased nodal properties (mainly located in the SFG, MTG, CG and PCun) and functional connectivities (mainly between anterior DMN and posterior DMN), which further strengthened the evidence of DMN dissociation in ESRD patients. Moreover, the average functional connectivity between anterior DMN and posterior DMN (aDMN-pDMN) in ESRD patients was positively correlated with TNF-alpha, which proved the association between DMN disassociation and systemic inflammation. Particularly, several increased nodal properties and functional connectivities were also observed in our study, which could be interpreted as compensatory mechanism for maintaining some aspect of cognition (e.g. MoCA and LTT test). Taken together, these findings implied a deviation from optimized cost-effective configuration of the DMN in ESRD patients. The DMN is critical for maintaining internal mentation and self-directed thought 49. The disrupted connectivities may reflect a reduced ability to maintain normal cognitive function in ESRD patients.

As for the analysis of inflammatory status, we found increased inflammatory cytokine level (IL-6, IFN-gamma and TNF-alpha) in ESRD patients. The TNF-alpha was correlated with emotion scale sore (SDS) and global network parameters (aDMN-pDMN, aGamma and aSigma). Previous studies have also reported that TNF-alpha was involved in the development of inflammation-associated depressive disorder 50,51. In this study, Parabacteroides was correlated with TNF-alpha and SDS, meanwhile, Parabacteroides and Megasphaera were correlated with TNF-alpha and SAS. These results may hint the inner relationship among “gut-inflammation-brain” to some extent. Furthermore, using mediation analyses, we confirmed the following pathway: reduction of butyrate producing bacteria (less abundance of Roseburia) leads to DMN dissociation (decreased aDMN-pDMN connectivity) via increasing inflammatory cytokine (higher level of IL-6) in patients with ESRD. In the gut microbiota analysis, Roseburia was found to be significantly less abundant in ESRD patients than HCs, which was consisting with previous findings 52. Roseburia is one of the dominant intestinal bacterial microbiota, which could induce anti-inflammatory responses by regulating Treg cell 53. Some studies have also reported that, in subjects with CKD, reduction of the Roseburia (a butyrate producing bacteria) could contribute to micro-inflammatory status and disease progression 52. These results, in conjunction with other studies 54, indicate that butyrate producing bacteria shows the potential to be new probiotics in the treatment of ESRD patients.

Here, we firstly revealed the ESRD-based inflammation mediating pathway linking the gut microbiota to brain changes: gut microbiota alteration impairs patients' brain by increasing systemic inflammation. However, causation from this observed mediating pathway should be cautiously inferred, given the nature of our cross-sectional comparison in the present study.

There are several strengths in this study. First, to the best of our knowledge, this is the first study which simultaneously investigated the gut microbiota, inflammatory cytokines, brain network and cognitive ability, and also further explored the potential linkages between these factors. To some extent, the novelty of combining functional brain network, cognitive function, gut microbiota and inflammatory state is prominent. Second, as we know, some previous studies which detected the DMN changes only selected small numbers of nodes. The reliance on a small number of nodes could have resulted in a decreased sensitivity to detect the differences. By adopting a 50-nodes network for the DMN, we have taken a step towards more accurate analysis of DMN. Third, we repeated the network analysis using another functional brain atlas (Power 264 atlas) to ensure the reliability of network analysis results.

There are also several limitations which warrant attention and suggest directions for future research. First, this study is cross-sectional design, which prevents us to exactly figure out the causal relationship between these factors. Further longitudinal studies with intestinal flora intervention are urgently needed. Secondly, utilizing multiple imaging modalities (such as diffusion tensor imaging) to explore the structural substrate underlying the DMN topological abnormality would be beneficial. Third, we only revealed the small-world properties of the DMN, but patients with ESRD may exhibit disruptions in other brain networks, such as the executive control network and emotion network. Therefore, further investigations of other specific brain networks are necessary. Fourthly, the sample size of our study is relatively small, the statistical power may be limited, However, we have noticed that some published articles which conducted microbiota mediation analysis had similar sample size (n=about 20-40) as ours. Hence, though sample size is limited, the findings of this study should be reliable and may provide insight of this topic. Finally, some other confounding factors may affect the results although we considered many confounding factors in this prospective pilot study with small sample size. Thus, it is needed to further collect a large sample size in future.

## Conclusion

In the current study, ESRD patients showed gut microbiota alterations, systemic inflammation, DMN dissociation and cognitive dysfunction. Importantly, inflammatory cytokine mediated the effect of gut microbiota on the brain in the context of ESRD, which indicated the potential role of probiotics in the treatment of ESRD-induced cognitive disorders.

## Supplementary Material

Supplementary figures and tables.

## Figures and Tables

**Table 1:** Demographics, biochemistry tests, inflammatory cytokines and neuropsychological assessments between ESRD and HC

	ESRD (n=28)	HC (n=19)	P value
Demographics			
Age (y)	43.9 ± 13.8	44.1 ± 10.0	0.954^c^
Gender (M/F)	13/15	12/7	0.382^b^
Education (y)	11.1 ± 3.6	12.4 ± 4.2	0.271^c^
BMI (kg/m^2^)	20.6 ± 2.6	23.7 ± 3.1	0.001^c^
TIV (cm^3^)	1133.4 ± 134	1202.7 ± 116	0.072^c^
Biochemistry indicator			
Urea nitrogen (mg/dl)	54.1 ± 24.6	-	-
Serum creatinine (mg/dl)	9.09 ± 3.90	-	-
Uric acid (mg/dl)	359.9 ± 172.0	-	-
eGFR (ml/min/1.73m^2^)	5.0 (9.0 - 4.0)	-	-
Inflammation cytokines			
IL-6 (pg/mL)	1.7 ± 0.7	0.9 (1.1 - 0.7)	0.002^a^
IL-1alpha (pg/mL)	0.2 ± 0.1	0.1 ± 0.1	0.075^c^
IFN-gamma (pg/mL)	0.7 ± 0.5	0.2 (0.6 - 0.1)	0.028^a^
TNF-alpha (pg/mL)	0.6 ± 0.4	0.2 ± 0.2	0.000^c^
Neuropsychological assessments			
MMSE/30 (score)	29 (30 - 28)	29 (30 - 28)	0.999^ a^
MoCA/30 (score)	25.5 (27.0 - 25.0)	28 (29 - 26)	0.005^a^
NCT-A (s)	60.0 (79.5 - 40.0)	44.5 ± 8.4	0.036^a^
LTT (s)	59.2 ± 19.8	48.6 ± 16.3	0.060^c^
SDT (s)	49.2 ± 13.4	37.3 ± 5.4	0.001^c^
DST (n)	37.8 ± 12.7	48.9 ± 15.4	0.010^c^
SAS (score)	31.2 ± 5.2	27.4 ± 4.5	0.007^ c^
SDS (score)	35.6 ± 7.2	28.6 ± 6.3	0.001^c^

a, Mann-Whitney U test; b, χ^2^ test; c, independent two sample t-test. eGFR= estimated glomerular filtration rate; ESRD: end-stage renal disease; F: female; HC: healthy control; IFN: interferon; IL: interleukin; LTT: line-tracing test; M: male; MMSE: mini-mental state examination; MoCA: Montreal cognitive assessment; n: number; NCT: number connection test; s: second; SAS: self-rating anxiety scale; SDS: self-rating depression scale; SDT: serial dotting test; TNF: tumor necrosis factor; y: year.

**Table 2:** Brain network graph measures in ESRD patients and HC

	ESRD (n=28)	HC (n=19)	P value
aDMN-pDMN	0.454 ± 0.177	0.645 ± 0.116	0.001
aGamma	0.673 ± 0.146	0.502 ± 0.712	0.003
aLambda	0.412 ± 0.021	0.424 ± 0.028	0.131
aSigma	0.516 ± 0.110	0.378 ± 0.062	0.001
aCp	0.107 ± 0.016	0.116 ± 0.014	0.011
aLp	1.317 ± 0.126	1.269 ± 0.069	0.110
aEg	0.081 ± 0.008	0.084 ± 0.004	0.078
aEloc	0.126 ± 0.015	0.134 ± 0.013	0.024

aCp: the area under curve of clustering coefficient; aDMN: anterior default mode network; aEg: the area under curve of global efficiency; aEloc: the area under curve of local efficiency; aGamma: the area under curve of Gamma; aLambda: the area under curve of Lambda; aLp: the area under curve of characteristic path length; aSigma: the area under curve of Sigma; ESRD: end-stage renal disease; HC: healthy control; pDMN: posterior default mode network.

## Abbreviations

DMN: default mode network
ESRD: end-stage renal disease
FC: functional connectivity
HC: healthy control
IL-6: interleukin-6
