# Supplementary material for: The gut microbiota-inflammation-brain axis in end-stage renal disease: perspectives from default mode network
Source: Theranostics. 2019 Oct 18;9(26):8171–81. doi: 10.7150/thno.35387 (PMC6857049; doi:10.7150/thno.35387)
Supplement: Supplementary file 1 — Supplementary figures and tables. [file thnov09p8171s1.pdf]

Supplementary Materials:

Scanning parameters and preprocessing steps:

The scanning parameters of rs-fMRI: repetition time (TR) = 2,000 ms, echo time (TE) = 30 ms, field of view (FOV) = 240×240 mm^2, flip angle = 90°, acquisition matrix = 64 × 64, 250 time points, slice thickness = 3 mm, slices = 43, 250 volumes and 30 axial slices for whole-brain coverage.

The scanning parameters of 3D-T1WI: TR = 1,900 ms, TE = 2.26 ms, FOV = 250×250 mm^2, slice thickness = 1.0 mm, gap = 0.5 mm, flip angle = 9°, resolution matrix = 256 × 256 and 176 slices in the sagittal orientation.

Rs-fMRI preprocessing included the following 6 steps: (1) the first 10 volumes of each fMRI image were discarded for the signal equilibrium/scanner stabilization and participants' adaptation to the scanning noise; (2) slice timing, realignment, and head motion correction were performed for the remaining 240 volumes; (3) high-resolution 3D-T1WI data were co-registered to the mean realigned functional images for each individual, and the transformed T1 structural images were segmented into gray matter, white matter, and cerebrospinal fluid. The realigned functional volumes were spatially normalized to the Montreal Neurological Institute (MNI) space and then each voxel was re-sampled to 3×3×3 mm^3; (4) the images were spatially smoothed with a 6-mm full-width at half-maximum Gaussian kernel; (5) the functional images were further linearly detrended, and temporal band-pass filtering (0.01-0.08 Hz) was performed to reduce the effect of physiological high-frequency noise and low-frequency drifts; (6) to reduce the effects of confounding factors, the nuisance signal (white matter, cerebrospinal fluid, and global signal) and the Friston 24-parameter model was eliminated by regression analyses.

Gut microbiota analysis procedures:

DNA samples were quantified and validated by Qubit 2.0 (Invitrogen, Carlsbad, CA, USA), and then the V3-V4 hypervariable region of 16S rRNA was selected as a target fragment for amplification and sequencing. PCR mixture was configured for PCR amplification, and PCR product was selected by agarose gel electrophoresis to select a specific length band, tapping and recovering the purified target band. The same amount of amplification solution was conducted with Illumina MiSeq sequencing with a double-ended PE300 strategy following the instrument manual (Illumina, San Diego, CA, USA). Image analysis and data conversion processes were computed by using the instrument's own software, MiSeq Control Software (2.5.0.5).

The raw reads were filtered to remove low quality and polyclonal sequences in QIIME (Version 1.9.1). The filtered data were further compared with the Gold database and the chimera reads were detected by using Uchime algorithm in Usearch software (Version 8.1.1861). And the effective tags were clustered using the Uclust method of QIIME software (Version 1.9.1) according to Operational Taxonomic Units with 97% similarity. Each OTU representative sequence was selected for species annotation and classification by using QIIME software (Version 1.9.1) and Silva database (Release 132). The Alpha diversity Shannon index was calculated by using QIIME software (Version 1.9.1).

The species difference analysis at all species levels were performed based on the bacterial population difference statistics of the fecal samples between the two groups by the Linear Discriminant Analysis Effect Size software. Based on the information on the abundance distribution of the horizontal species, the Wilcoxon signed rank test was performed between the two groups, and the genus species with significant differences between the two groups were obtained and the abundance distribution map was drawn.

Mediation analysis steps:

According to the standard conventions of a mediation analysis, the test comprises four steps: (1) path c: the effect of the gut microbiota on DMN topological alterations, namely, the total effect of the predictors on the outcome; (2) path a: the effect of the gut microbiota on

systematic inflammation; (3) path b: the relationship between systematic inflammation and DMN topological alterations after controlling for the gut microbiota; and (4) the a×b effect, which was referred to as the indirect effect and indicates whether the predictor-outcome relationship was significantly reduced after controlling for the mediator. In the interest of minimizing the number of statistical comparisons, only the indexes displaying a significant difference between the ESRD and HC groups were entered into this mediation model as candidate mediators. In these tests, age and sex were also included as covariates.

Supplementary Figures and Figure Legends

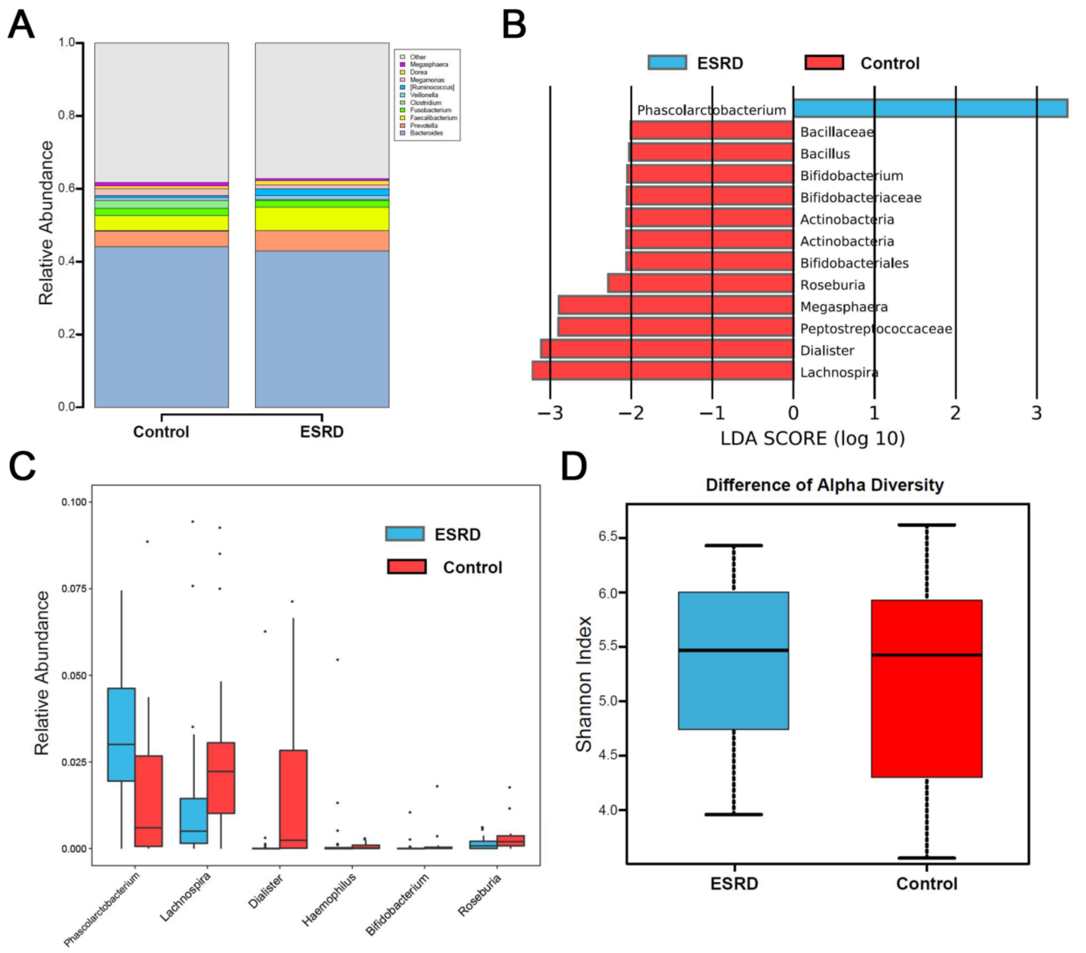

Figure S1. Analysis of Gut Microbiota in Patients with ESRD

Panel A shows that ESRD and the HC groups were mainly abundant with Bacteroides. **Panel B** is the species abundance LEfSe difference analysis. The ESRD group was mainly enriched in the Phascolarctobacterium, while HC group was mainly enriched in the Roseburia, Megasphaera, Peptostreptococcaceae, Dialister, Lachnospira, Bifidobacteriales, Bifidobacteriaceae, Bifidobacterium, Bacillaceae, Bacillus and Actinobacteria. **Panel C** is the Wilcox differential analysis based on genus-level species abundancies. The ESRD group was mainly enriched in Phascolarctobacterium, Holdemania and Eggerthella, while mainly decreased in Roseburia, Lachnospira, Dialister and Bifidobacterium. **Panel D** is the Alpha diversity analysis of microflora. The differences of intestinal flora alpha diversity between the ESRD group and HC group was not significant ($p = 0.497$) according to the Shannon index.

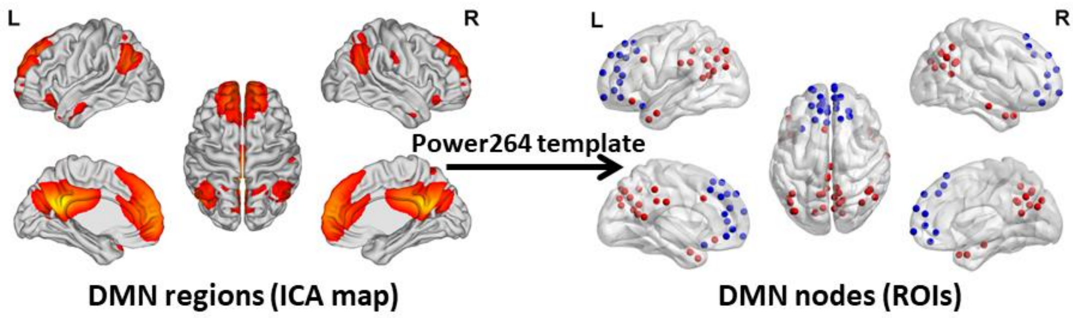

Figure S2. DMN regions and DMN Nodes using Power 264 Atlas

The graphs depict the 55 DMN nodes derived from the functional atlas (Power 246) that were used for the primary analysis. The picture on the left side shows the DMN map and the picture on the right side shows the ROI selected according to the DMN map. The blue ROIs represent the regions belong to the anterior DMN and the red ROIs represent the regions belong to the posterior DMN. The picture is made using the BrainNet Viewer software (http://www.nitrc.org/projects/bnv).

DMN: default mode network; ICA: independent component analysis; ROI: region of interest

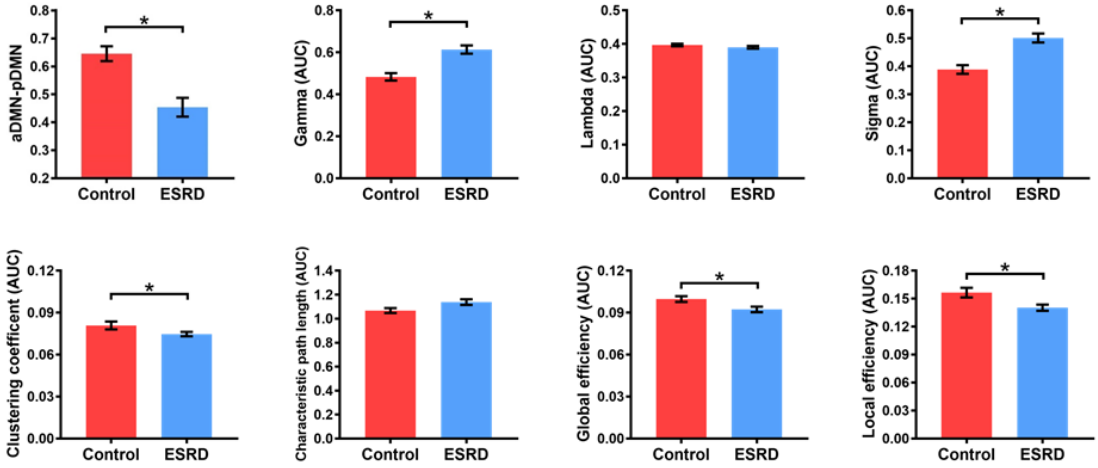

Figure S3. Differences in Global Network Measures of the DMN using Power 264 Atlas

The graphs show global network measures of the DMN. The red histogram represents healthy controls and the blue histogram represents ESRD patients. Black asterisks (*) indicate the significant difference between groups ($p < 0.05$).

DMN: default mode network; ESRD: end-stage renal disease; AUC: area under the curve

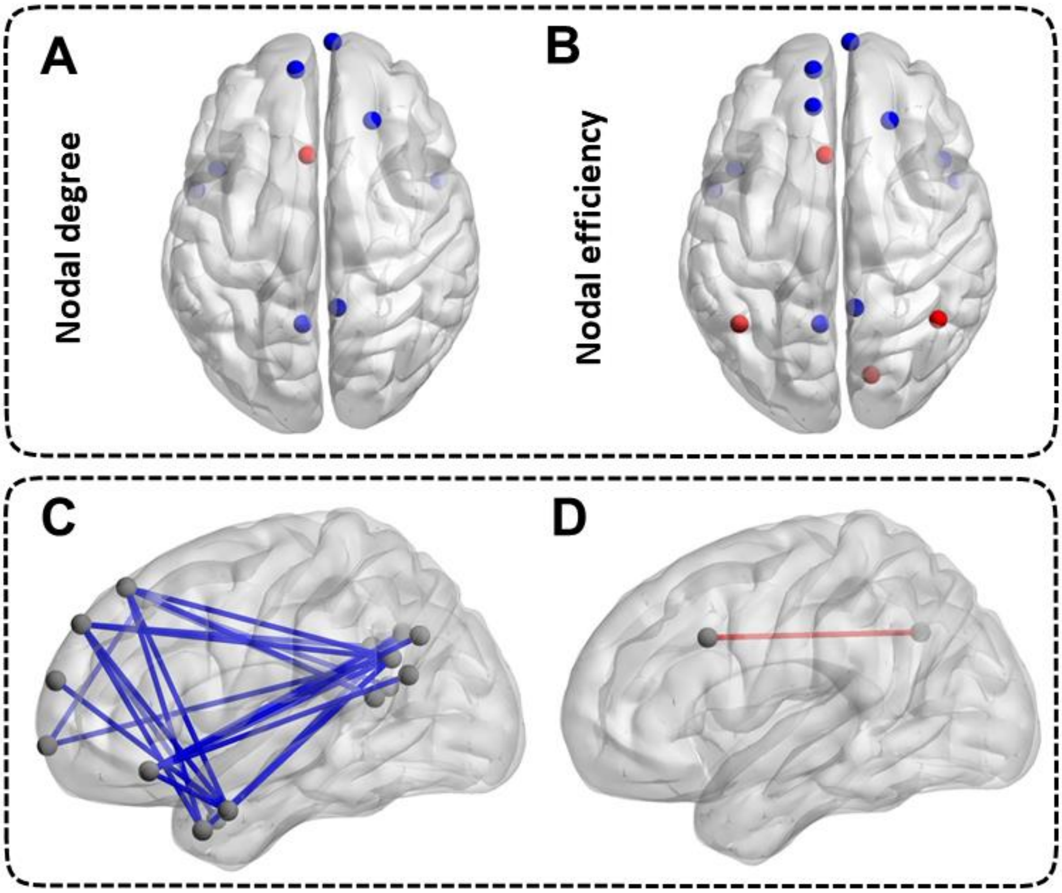

Figure S4. Differences in Nodal Network Measures and Functional Connectivity Measures of the DMN using Power 264 Atlas

The top half of the picture represents the differences in nodal network measures, while the bottom half of the picture represents the differences in functional connectivity measures. The blue nodes and blue lines indicate decreased nodal centralities and functional connectivities. The red nodes and red lines indicate increased nodal centralities and functional connectivities.

Supplementary Tables

Table S1. Detailed information of the BN246 template and default mode network (DMN) regions selected in this study

Gyrus	Left and Right Hemisphere	Label ID.L	Label ID.R	lh.MNI(X,Y,Z)	rh.MNI(X,Y,Z)
SFG, Superior Frontal Gyrus	SFG_L(R)_7_1	1	2	-5 ,15, 54	7, 16, 54
	SFG_L(R)_7_2	3	4	-18, 24, 53	22, 26, 51
	SFG_L(R)_7_3	5	6	-11, 49, 40	13, 48, 40
	SFG_L(R)_7_4	7	8	-18, -1, 65	20, 4, 64
	SFG_L(R)_7_5	9	10	-6, -5, 58	7, -4, 60
	SFG_L(R)_7_6	11	12	-5, 36, 38	6, 38, 35
	SFG_L(R)_7_7	13	14	-8, 56, 15	8, 58, 13
MFG, Middle Frontal Gyrus	MFG_L(R)_7_1	15	16	-27, 43, 31	30, 37, 36
	MFG_L(R)_7_2	17	18	-42, 13, 36	42, 11, 39
	MFG_L(R)_7_3	19	20	-28, 56, 12	28, 55, 17
	MFG_L(R)_7_4	21	22	-41, 41, 16	42, 44, 14
	MFG_L(R)_7_5	23	24	-33, 23, 45	42, 27, 39
	MFG_L(R)_7_6	25	26	-32, 4, 55	34, 8, 54
	MFG_L(R)_7_7	27	28	-26, 60, -6	25, 61, -4
IFG, Inferior Frontal Gyrus	IFG_L(R)_6_1	29	30	-46, 13, 24	45, 16, 25
	IFG_L(R)_6_2	31	32	-47, 32, 14	48, 35, 13
	IFG_L(R)_6_3	33	34	-53, 23, 11	54, 24, 12
	IFG_L(R)_6_4	35	36	-49, 36, -3	51, 36, -1

	IFG_L(R)_6_5	37	38	-39, 23, 4	42, 22, 3
	IFG_L(R)_6_6	39	40	-52, 13, 6	54, 14, 11
OrG, Orbital Gyrus	OrG_L(R)_6_1	41	42	-7, 54, -7	6, 47, -7
	OrG_L(R)_6_2	43	44	-36, 33, -16	40, 39, -14
	OrG_L(R)_6_3	45	46	-23, 38, -18	23, 36, -18
	OrG_L(R)_6_4	47	48	-6, 52, -19	6, 57, -16
	OrG_L(R)_6_5	49	50	-10, 18, -19	9, 20, -19
	OrG_L(R)_6_6	51	52	-41, 32, -9	42, 31, -9
PrG, Precentral Gyrus	PrG_L(R)_6_1	53	54	-49, -8, 39	55, -2, 33
	PrG_L(R)_6_2	55	56	-32, -9, 58	33, -7, 57
	PrG_L(R)_6_3	57	58	-26, -25, 63	34, -19, 59
	PrG_L(R)_6_4	59	60	-13, -20, 73	15, -22, 71
	PrG_L(R)_6_5	61	62	-52, 0, 8	54, 4, 9
	PrG_L(R)_6_6	63	64	-49, 5, 30	51, 7, 30
PCL, Paracentral Lobule	PCL_L(R)_2_1	65	66	-8, -38, 58	10, -34, 54
	PCL_L(R)_2_2	67	68	-4, -23, 61	5, -21, 61
STG, Superior Temporal Gyrus	STG_L(R)_6_1	69	70	-32, 14, -34	31, 15, -34
	STG_L(R)_6_2	71	72	-54, -32, 12	54, -24, 11
	STG_L(R)_6_3	73	74	-50, -11, 1	51, -4, -1
	STG_L(R)_6_4	75	76	-62, -33, 7	66, -20, 6
	STG_L(R)_6_5	77	78	-45, 11, -20	47, 12, -20
	STG_L(R)_6_6	79	80	-55, -3, -10	56, -12, -5

MTG, Middle Temporal Gyrus	MTG_L(R)_4_1	81	82	-65, -30, -12	65, -29, -13
	MTG_L(R)_4_2	83	84	-53, 2, -30	51, 6, -32
	MTG_L(R)_4_3	85	86	-59, -58, 4	60, -53, 3
	MTG_L(R)_4_4	87	88	-58, -20, -9	58, -16, -10
ITG, Inferior Temporal Gyrus	ITG_L(R)_7_1	89	90	-45, -26, -27	46, -14, -33
	ITG_L(R)_7_2	91	92	-51, -57, -15	53, -52, -18
	ITG_L(R)_7_3	93	94	-43, -2, -41	40, 0, -43
	ITG_L(R)_7_4	95	96	-56, -16, -28	55, -11, -32
	ITG_L(R)_7_5	97	98	-55, -60, -6	54, -57, -8
	ITG_L(R)_7_6	99	100	-59, -42, -16	61, -40, -17
	ITG_L(R)_7_7	101	102	-55, -31, -27	54, -31, -26
FuG, Fusiform Gyrus	FuG_L(R)_3_1	103	104	-33, -16, -32	33, -15, -34
	FuG_L(R)_3_2	105	106	-31, -64, -14	31, -62, -14
	FuG_L(R)_3_3	107	108	-42, -51, -17	43, -49, -19
PhG, Parahippocampal Gyrus	PhG_L(R)_6_1	109	110	-27, -7, -34	28, -8, -33
	PhG_L(R)_6_2	111	112	-25, -25, -26	26, -23, -27
	PhG_L(R)_6_3	113	114	-28, -32, -18	30, -30, -18
	PhG_L(R)_6_4	115	116	-19, -12, -30	19, -10, -30
	PhG_L(R)_6_5	117	118	-23, 2, -32	22, 1, -36
	PhG_L(R)_6_6	119	120	-17, -39, -10	19, -36, -11
pSTS, posterior Superior Temporal Sulcus	pSTS_L(R)_2_1	121	122	-54, -40, 4	53, -37, 3
	pSTS_L(R)_2_2	123	124	-52, -50, 11	57, -40, 12

SPL, Superior Parietal Lobule	SPL_L(R)_5_1	125	126	-16, -60, 63	19, -57, 65
	SPL_L(R)_5_2	127	128	-15, -71, 52	19, -69, 54
	SPL_L(R)_5_3	129	130	-33, -47, 50	35, -42, 54
	SPL_L(R)_5_4	131	132	-22, -47, 65	23, -43, 67
	SPL_L(R)_5_5	133	134	-27, -59, 54	31, -54, 53
IPL, Inferior Parietal Lobule	IPL_L(R)_6_1	135	136	-34, -80, 29	45, -71, 20
	IPL_L(R)_6_2	137	138	-38, -61, 46	39, -65, 44
	IPL_L(R)_6_3	139	140	-51, -33, 42	47, -35, 45
	IPL_L(R)_6_4	141	142	-56, -49, 38	57, -44, 38
	IPL_L(R)_6_5	143	144	-47, -65, 26	53, -54, 25
	IPL_L(R)_6_6	145	146	-53, -31, 23	55, -26, 26
Pcun, Precuneus	PCun_L(R)_4_1	147	148	-5, -63, 51	6, -65, 51
	PCun_L(R)_4_2	149	150	-8, -47, 57	7, -47, 58
	PCun_L(R)_4_3	151	152	-12, -67, 25	16, -64, 25
	PCun_L(R)_4_4	153	154	-6, -55, 34	6, -54, 35
PoG, Postcentral Gyrus	PoG_L(R)_4_1	155	156	-50, -16, 43	50, -14, 44
	PoG_L(R)_4_2	157	158	-56, -14, 16	56, -10, 15
	PoG_L(R)_4_3	159	160	-46, -30, 50	48, -24, 48
	PoG_L(R)_4_4	161	162	-21, -35, 68	20, -33, 69
INS, Insular Gyrus	INS_L(R)_6_1	163	164	-36, -20, 10	37, -18, 8
	INS_L(R)_6_2	165	166	-32, 14, -13	33, 14, -13
	INS_L(R)_6_3	167	168	-34, 18, 1	36, 18, 1

	INS_L(R)_6_4	169	170	-38, -4, -9	39, -2, -9
	INS_L(R)_6_5	171	172	-38, -8, 8	39, -7, 8
	INS_L(R)_6_6	173	174	-38, 5, 5	38, 5, 5
CG, Cingulate Gyrus	CG_L(R)_7_1	175	176	-4, -39, 31	4, -37, 32
	CG_L(R)_7_2	177	178	-3, 8, 25	5, 22, 12
	CG_L(R)_7_3	179	180	-6, 34, 21	5, 28, 27
	CG_L(R)_7_4	181	182	-8, -47, 10	9, -44, 11
	CG_L(R)_7_5	183	184	-5, 7, 37	4, 6, 38
	CG_L(R)_7_6	185	186	-7, -23, 41	6, -20, 40
	CG_L(R)_7_7	187	188	-4, 39, -2	5, 41, 6
MVOcC, MedioVentral Occipital Cortex	MVOcC _L(R)_5_1	189	190	-11, -82, -11	10, -85, -9
	MVOcC _L(R)_5_2	191	192	-5, -81, 10	7, -76, 11
	MVOcC _L(R)_5_3	193	194	-6, -94, 1	8, -90, 12
	MVOcC _L(R)_5_4	195	196	-17, -60, -6	18, -60, -7
	MVOcC _L(R)_5_5	197	198	-13, -68, 12	15, -63, 12
LOcC, lateral Occipital Cortex	LOcC_L(R)_4_1	199	200	-31, -89, 11	34, -86, 11
	LOcC _L(R)_4_2	201	202	-46, -74, 3	48, -70, -1
	LOcC _L(R)_4_3	203	204	-18, -99, 2	22, -97, 4
	LOcC_L(R)_4_4	205	206	-30, -88, -12	32, -85, -12
	LOcC _L(R)_2_1	207	208	-11, -88, 31	16, -85, 34
	LOcC _L(R)_2_2	209	210	-22, -77, 36	29, -75, 36
Amyg, Amygdala	Amyg_L(R)_2_1	211	212	-19, -2, -20	19, -2, -19

	Amyg_L(R)_2_2	213	214	-27, -4, -20	28, -3, -20
Hipp, Hippocampus	Hipp_L(R)_2_1	215	216	-22, -14, -19	22, -12, -20
	Hipp_L(R)_2_2	217	218	-28, -30, -10	29, -27, -10
BG, Basal Ganglia	BG_L(R)_6_1	219	220	-12, 14, 0	15, 14, -2
	BG_L(R)_6_2	221	222	-22, -2, 4	22, -2, 3
	BG_L(R)_6_3	223	224	-17, 3, -9	15, 8, -9
	BG_L(R)_6_4	225	226	-23, 7, -4	22, 8, -1
	BG_L(R)_6_5	227	228	-14, 2, 16	14, 5, 14
	BG_L(R)_6_6	229	230	-28, -5, 2	29, -3, 1
Tha, Thalamus	Tha_L(R)_8_1	231	232	-7, -12, 5	7, -11, 6
	Tha_L(R)_8_2	233	234	-18, -13, 3	12, -14, 1
	Tha_L(R)_8_3	235	236	-18, -23, 4	18, -22, 3
	Tha_L(R)_8_4	237	238	-7, -14, 7	3, -13, 5
	Tha_L(R)_8_5	239	240	-16, -24, 6	15, -25, 6
	Tha_L(R)_8_6	241	242	-15, -28, 4	13, -27, 8
	Tha_L(R)_8_7	243	244	-12, -22, 13	10, -14, 14
	Tha_L(R)_8_8	245	246	-11, -14, 2	13, -16, 7

This table illustrates the detailed information of the BN246 template, more descriptions please see http://atlas.brainnetome.org

The regions highlighted in blue represent anterior DMN regions; the regions highlighted in red represent posterior DMN regions.

Table S2. Descriptions of the graph measures

Graph measure	Definition	Meaning
Clustering coefficient	quantifies the number of connections that exist between the nearest neighbors of a region as a proportion of the maximum number of possible connections	a measure of local network connectivity (i.e. a network with a high clustering coefficient is characterized by densely connected local clusters)
Characteristic path length	the minimum number of connections that must be traversed from one region to another region	a measure of global network connectivity (i.e. a network with low characteristic path length is characterized by short distances between two nodes)
Gamma	normalized clustering coefficient (compared to 100 random network)	imply network segregation of network
Lambda	normalized characteristic path length (compared to 100 random network)	imply network integration of the network
Sigma	the proportion of Gamma and Lambda (compared to 100 random networks)	evaluate the balance of segregation and integration of the network
Global efficiency	the average of inverse shortest path length of the whole network, which is inversely related to the characteristic path length	reflects the average efficiency of global network, represents the capacity of parallel information transmission over the network
Local efficiency	the average of the global efficiency of the sub-networks computed on the nearest neighbors of a region, which is related to the clustering coefficient	reflects the average efficiency of local network, represents the capacity of a network to transmit information at the local level and measures the fault tolerance of the network
Nodal degree	the number of edges of a node that connect with the remaining nodes in the network	measures how interactive a particular node is in the network
Nodal efficiency	the inverse of the harmonic mean of the shortest path length in the network	quantifies the importance of the nodes for communication within the network
Nodal betweenness	the fraction of shortest paths between two nodes passing through the area in the network	measures the influence of a region on network communication

Table S3. Significant Correlation between Gut Microbiota, Inflammatory Cytokines, Cognitive Assessments and DMN parameters in ESRD Group

**Factor1**	**Factor2**	**r value**	**P value**
aDMN-pDMN	MoCA	0.363	0.012
aDMN-pDMN	TNFα	-0.354	0.015
aDMN-pDMN	Vogesella	-0.463	0.046
aGamma	Odoribacter	0.324	0.026
aGamma	Selenomonas	0.309	0.034
aGamma	Schwartzia	0.309	0.034
aGamma	Syntrophus	0.304	0.038
aGamma	MoCA	0.356	0.014
aGamma	NCT	0.396	0.006
aGamma	DST	-0.337	0.021
aGamma	SDT	0.443	0.002
aGamma	TNFα	0.410	0.004
aSigma	MoCA	0.378	0.009
aSigma	NCT	0.366	0.011
aSigma	DST	-0.318	0.029
aSigma	SDT	0.454	0.001
aSigma	SAS	0.307	0.036
aSigma	TNFα	0.360	0.013
aCp	Collinsella	-0.394	0.006
aCp	Coprobacillus	-0.350	0.016
aCp	Prevotella	-0.346	0.017
aCp	Comamonas	-0.343	0.018
aCp	Epulopiscium	-0.302	0.039
aCp	Helicobacter	-0.302	0.039
aEloc	Collinsella	-0.356	0.014
aEloc	Coprobacillus	-0.351	0.016
aEloc	Comamonas	-0.331	0.023
aEloc	Prevotella	-0.311	0.034
MoCA	Lachnospira	-0.472	0.041
MoCA	cc_115	-0.520	0.022
MoCA	Candidatus_Rhodoluna	-0.584	0.009
MoCA	Sediminibacterium	-0.520	0.022
MoCA	Finegoldia	-0.520	0.022
NCT	Prevotella	0.479	0.038
NCT	Paraprevotella	0.508	0.026

NCT	Slackia	-0.496	0.031
NCT	Bacillus	0.498	0.030
SDT	Anaerofustis	-0.459	0.048
DST	Clostridium	-0.474	0.040
DST	Granulicatella	-0.461	0.047
DST	Rothia	-0.463	0.046
DST	Oribacterium	-0.461	0.047
DST	Moryella	-0.456	0.050
DST	Actinobacillus	-0.477	0.039
DST	Actinomyces	-0.499	0.030
DST	Parvimonas	-0.550	0.015
DST	Peptostreptococcus	-0.456	0.050
DST	Bulleidia	-0.456	0.050
DST	Cardiobacterium	-0.456	0.050
DST	Chryseobacterium	-0.456	0.050
DST	SMB53	-0.461	0.047
DST	Methylobacterium	-0.456	0.050
DST	TG5	-0.456	0.050
DST	Peptoniphilus	-0.456	0.050
SAS	Megasphaera	0.536	0.018
SAS	Parabacteroides	0.654	0.002
SDS	Parabacteroides	0.472	0.041
SDS	Paraprevotella	-0.484	0.036
TNFα	SDS	0.417	0.004
TNF-α	Veillonella	0.506	0.027
TNF-α	Megasphaera	0.484	0.036
TNF-α	Parabacteroides	0.552	0.014
IFN-γ	Oscillospira	-0.503	0.047
IFN-γ	Acidaminococcus	0.668	0.005
IL-6	Coprococcus	0.613	0.009
IL-6	Dialister	0.655	0.004
IL-6	Anaerofustis	0.767	0.001

Table S4. Brain network graph measures in ESRD patients and HC using Power 264 Atlas

	ESRD (n=28)	HC (n=19)	P value
aDMN-pDMN	0.454 ± 0.177	0.645 ± 0.116	**0.001**
aGamma	0.613 ± 0.103	0.483 ±0.078	**0.002**
aLambda	0.390 ± 0.021	0.397 ± 0.017	0.238
aSigma	0.501 ± 0.085	0.389 ± 0.067	**0.002**
aCp	0.075 ± 0.008	0.081 ± 0.012	**0.003**
aLp	1.138 ± 0.125	1.067 ± 0.089	0.065
aEg	0.092 ± 0.010	0.100 ± 0.009	**0.015**
aEloc	0.140 ± 0.018	0.156 ± 0.022	**0.009**

aCp: the area under curve of clustering coefficient; aDMN: anterior default mode network; aEg: the area under curve of global efficiency; aEloc: the area under curve of local efficiency; aGamma: the area under curve of Gamma; aLambda: the area under curve of Lambda; aLp: the area under curve of characteristic path length; aSigma: the area under curve of Sigma; ESRD: end-stage renal disease; HC: healthy control; pDMN: posterior default mode network

Power 264 Atlas refers to Power JD, Mitra A, Laumann TO, Snyder AZ, Schlaggar BL, Petersen SE. Methods to detect, characterize, and remove motion artifact in resting state fMRI. Neuroimage. 2014; 84: 320-41.
